# Collagen II antibody-induced arthritis in Tg1278TNFko mice: optimization of a novel model to assess treatments targeting human TNFα in rheumatoid arthritis

**DOI:** 10.1186/s12967-014-0285-z

**Published:** 2014-10-25

**Authors:** Adrian Richard Moore, Sarah Allden, Tim Bourne, Maria C Denis, Ksanthi Kranidioti, Remi Okoye, Yannis Sotsios, Zofia Stencel, Alexander Vugler, Gillian Watt, Stevan Shaw

**Affiliations:** UCB Celltech, 208 Bath Road, Slough, Berkshire SL1 3WE UK; Biomedcode Hellas SA, 34 Al. Fleming St., 166 72, Vari, Hellas Greece

**Keywords:** Collagen antibody induced arthritis, Rheumatoid arthritis, Tg1278TNFko mice, TNFα

## Abstract

**Background:**

Novel molecules that specifically target human TNFα in rheumatoid arthritis pose problems for preclinical assessment of efficacy. In this study collagen antibody-induced arthritis (CAIA) has been induced in human TNFα transgenic mice to provide a novel model that has been optimised for the evaluation of molecules targeting human TNFα.

**Methods:**

Tg1278TNFko mice lack murine TNFα and are heterozygous for multiple copies of the human TNFα transgene that is expressed under normal physiological control. To establish CAIA, a collagen II monoclonal antibody cocktail (CAb) at 2, 4 or 8 mg was injected i.p. on Day 0 followed by a lipopolysaccharide (LPS) boost (10 or 100 μg) i.p. on Day 1 or Day 4. Animals were assessed for arthritis symptoms using a clinical score, cytokine levels (human TNFα, IL-1β and IL-6) in sera and joints, and histopathology. The dependence of the model on human TNFα was determined by dosing animals with etanercept.

**Results:**

Tg1278TNFko animals treated with 2, 4 or 8 mg CAb on Day 0, with 100μg LPS on Day 4, had more severe arthritis and earlier symptoms than wild type animals at all doses of CAb tested. Subsequently it was found that the transgenic model did not require LPS at all for arthritis development but a lower dose of LPS (10 μg) was found necessary for reproducible and robust disease (close to 100% incidence, well-synchronised, with high arthritis scores). Furthermore the LPS challenge could be brought forward to Day 1 so that its’ actions to facilitate disease could be separated temporally from the arthritis phase (beginning about Day 4). Etanercept, administered immediately after the serum spike of cytokines associated with LPS had subsided, was able to dose-dependently inhibit arthritis development and this was associated with a marked protection of the joints histologically on Day 14. Etanercept was also able to reverse the signs of arthritis when given therapeutically allowing animals to be matched for disease burden before dosing begins.

**Conclusions:**

The features of CAIA in Tg1278TNFko animals make the model well-suited to testing the next generation of therapeutics that will target human TNFα in rheumatoid arthritis.

## Background

In recent years the treatment of rheumatoid arthritis (RA) has been transformed by the development of biologics targeting tumour necrosis factor alpha (TNFα). Response rates to treatment, as assessed by American College of Rheumatology (ACR) criteria, are typically in the region of 60% ACR20, 40% ACR50 and 20% ACR70 at 24 weeks of treatment [1]. However biologics are expensive and so are not as widely used as they might be. Typically patients will have failed conventional treatments such as methotrexate before being considered suitable for anti TNFα therapy. The problem with this is that anti TNFα treatment might be more effective if initiated early, and indeed current thinking is to identify and treat RA patients as quickly as possible in an attempt to induce long term remission [2]. Another problem with biologics is that they are inconvenient to administer when compared with conventional therapy.

One view of biological agents directed towards TNFα is that they have validated TNFα as a target for the treatment of RA but that they will be superseded by small molecules that act on the same pathway. These treatments may be cheaper, more convenient to administer and suitable as first line therapy either alone or in combination with other anti-rheumatic drugs such as methotrexate.

Despite the success of anti TNFs in the treatment of RA, most of the preclinical work directed against TNFα was to support sepsis as a disease indication [3]. Unfortunately anti TNFs proved to be a spectacular failure in sepsis. However, subsequent work in the human TNFα Tg197 transgenic mouse model of arthritis supported the therapeutic potential of anti-human TNFα in RA [4] that was later confirmed by a small trial in RA [5]. It is interesting to speculate on whether anti TNFs would ever have reached the clinic for RA if preclinical development had been through conventional models such as collagen induced arthritis (CIA) in rodents. Targeting TNFα, although effective to some extent in CIA, is rather less active than targeting IL-1β [6,7] which is actually the reverse of the clinical findings. CIA is also time consuming and suffers from variable incidence and severity. So CIA cannot be considered an ideal model for the testing of the next generation of TNFα inhibitors.

Because TNFα is an “effector cytokine”, playing a role in inflammation and joint destruction, a short term model that avoids the need for immune sensitization but establishes a role for TNFα in an inflammatory arthritis should offer advantages over CIA. One such model is the collagen II antibody induced arthritis (CAIA) model [8] (the K/BxN serum transfer model is another [9]). These models tend to be more robust than CIA and have been shown to have a TNFα component [10,11]. However, species differences between mice and human are a problem for biologics and potentially also for small molecules depending upon their mode of action.

Transgenic technology can help here. The Tg197 mouse is a human TNFα transgenic mouse that over expresses TNFα [4]. These animals develop spontaneous and progressive arthritis from about 3 weeks of age demonstrating that pathology associated with TNFα in RA can be recapitulated in the mouse. However, although the model has been used with success for the preclinical profiling of biologics that target human TNFα [12] there are some drawbacks. The animals are sick from an early age, and the chronicity of the model (up to 7 weeks) has implications for compound requirements particularly if dosing needs to be once or twice a day.

Another transgenic model is the Tg1278 mouse [4]. The Tg1278 contains ~50 copies of a 3.6 kb DNA fragment comprising the entire human TNFα coding sequences flanked by its own 5′ and 3′ regulatory sequences. The Tg1278 has been crossed to TNFα knock-out mice, resulting in a mouse strain (Tg1278TNFko) with normally regulated and expressed human TNFα in the absence of mouse TNFα. The human TNFα partially compensates for defects in lymphoid architecture seen in TNFα knockout mice [13] and the animals are healthy and do not develop spontaneous disease. The aim of this study was to characterise CAIA in these animals as a potential new model for investigating novel treatments targeting TNFα in RA.

## Materials and methods

### Materials

Arthritomab collagen II antibody cocktail (CAb)(Cat: CIA-MAB-2C, LOT: S1109002) and lipopolysaccharide (LPS) E.coli (055:B5, LOT: 20318A4) were from MDbioscience. Etanercept (Enbrel®) was from Pfizer.

### Animals

Mice were housed in specific pathogen free conditions with 3–4 animals per cage of 335 cm^2^ (Techniplast, UK) on a dust free plant fibre bedding (TierWohl, Germany) and maintained at 21-24°C, 55-65% humidity, 10–12 air changes per hour and on a 12 hour light dark cycle. Animals had access to a standard maintenance diet (1324 Diet-Rats/Mice, Altromin, Germany) and water *ad libitum*. Wild type C57BL/6J mice were obtained from Charles River Laboratories. To generate Tg1278TNFko animals, male stud mice heterozygous for Tg1278 and homozygous for TNFko (maintained in a C57BL/6 genetic background) were crossed with TNFko C57BL6 females. Their Tg1278TNFko heterozygous transgenic offspring were identified by tail DNA genotyping. Groups were matched as closely as possible for mean body weights. Male and female animals were used as indicated.

Experiments were conducted at Biomedcode and conformed with the Presidential Decree No 160/1991 Governmental Gazette No A’ 64 applicable in Greece, which is the implementation of the EEC Directive 86/609/EEC. Protocols were reviewed and approved by the Institutional Animal Ethical Committee at the Biomedical Sciences Research Centre “Al. Fleming”.

### CAIA induction

Mice were injected with CAb intraperitoneally (i.p.) at 2-8 mg per mouse as indicated. Where LPS was administered this was either Day 1 or Day 4 at 10 or 100 μg i.p. per animal. Blood samples were collected at indicated times from retro-orbital bleeds using heparin coated glass capillaries. Serum was prepared and stored at −70°C prior to analysis. Clinical development of arthritis in the paws was assessed by arthritis score, 0 normal, 1 erythema and mild swelling confined to the tarsals or ankle joint, 2 erythema and mild swelling extending from the ankle to the tarsals, 3 erythema and moderate swelling extending from the ankle to metatarsal joints, 4 erythema and severe swelling encompass the ankle, foot and digits, or ankylosis of the limb. Thus the maximum score an animal could attain was 16.

### Dosing

Animals were dosed with etanercept where indicated. The drug was diluted with sterile saline and administered at 10 mL/kg intravenously (i.v.) for the first dose and subcutaneously (s.c.) for subsequent doses. Sterile saline served as a vehicle control.

### Cytokine assays

Cytokine measurements were made on serum samples and paws of CAIA animals. Paws were dissected above ankle at different time points post LPS challenge. One hind paw per animal was used for protein extraction. Collected paws were crushed in liquid nitrogen using a pulverizer and the tissue amount obtained from each paw was weighed. 1.5 mL of tissue extraction buffer (Life technologies), containing phosphatase inhibitor cocktail (Roche, 1 tablet 10 mL of extract) and protease inhibitors cocktail set IV (1:100 dilution, Merck), were used per sample. Samples were vortexed and then centrifuged at 4°C 2100 rpm for 10 minutes (IEC Centra GP8R) to pellet the tissue debris and supernatants re-centrifuged at 4°C 2000 rpm for 15 minutes. Supernatants were collected and total protein concentration in samples was measured using the microplate procedure for BCA assay (Thermo Scientific) according to the manufacturer’s instructions and absorbance was determine using the BCA assay setting on a NanoDrop (extraction solution was used as a blank). All samples were stored at −80°C prior to cytokine assay. Human TNFα cytokine ultra-sensitive kit (MSD, Meso Scale Discovery), mouse IL-17 cytokine ultra-sensitive kit (MSD, Meso Scale Discovery) and mouse proinflammatory 7-plex ultra-sensitive assay detecting IL-1β, IL-10, Il-12p70, INF-ɣ, IL-6, KC, TNFα (MSD, Meso Scale Discovery) were used to measure cytokine concentration in serum and protein extracts. The MSDs were used according to manufacturer protocols.

### Histopathological examination

Mouse paws were harvested, fixed in 10% neutral-buffered formalin, decalcified in 10% formic acid for 4 days, embedded in paraffin, sectioned, and stained with either haematoxylin & eosin for general evaluation or toluidine blue for specific assessment of cartilage changes. The stained slides were scanned on a whole slide scanner (Nanozoomer 2.0-HT, Hamamatsu, Japan) to acquire whole slide imaging at × 20 magnification.

### Statistical analysis

GraphPad Prism software was used to analyse all data. The in vivo arthritis score data were calculated as area under curve. These data and the cytokine data were analysed by one-way analysis of variance (ANOVA) with Bonferroni post-test.

## Results

**Figure 1:**
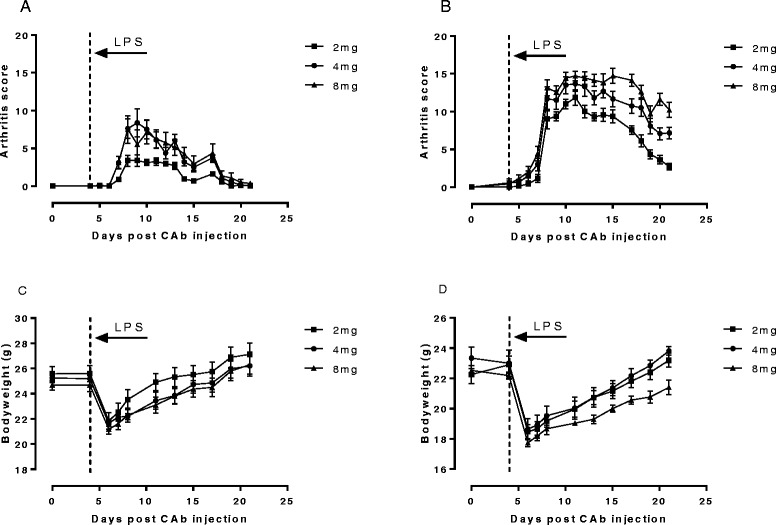
**CAIA in wild type and Tg1278TNFko animals.** Arthritis was induced in male mice by i.p. injection of 2, 4 or 8 mg collagen II antibody cocktail (CAb) on Day 0 followed by 100 μg LPS i.p. 4 days later. **A**, Arthritis scores of wild type animals. **B**, arthritis scores of Tg1278TNFko animals. **C**, Bodyweights of wild type animals. **D**, bodyweights of Tg1278TNFko animals. Data are means +/− s.e.m., n = 6-8/group.

Figure 1 shows arthritis score data and bodyweight data for wild type and Tg1278TNFko animals following induction of collagen antibody induced arthritis with 2, 4 or 8 mg CAb administered i.p. and 100 μg LPS administered i.p. 4 days later according to the manufacturer’s instructions. Incidence of arthritis in all groups was 100%, however, in wild type animals the severity of the arthritis was considerably lower than in the Tg1278TNFko animals for all doses of CAb administered.

LPS challenge was associated with weight loss that was evident from time of challenge (Day 4) to Day 6 when weights were next recorded. For wild type animals the weight loss amounted to approximately 16% but it was rather higher in the Tg1278TNFko animals at approximately 22%. Although bodyweights recovered as expected, the general condition of the Tg1278TNFko animals was a cause for concern throughout the experiment. A study was therefore conducted to see if a lower LPS challenge could be used in the Tg1278TNFko animals.

**Figure 2:**
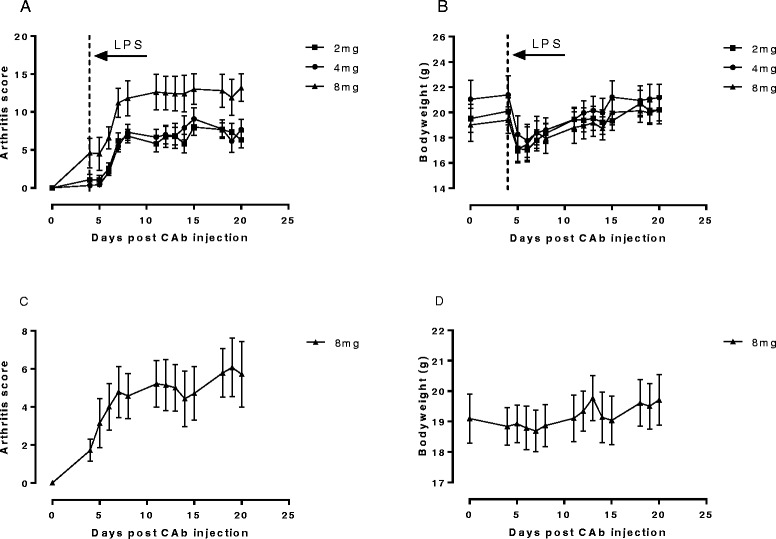
**CAIA in Tg1278TNFko animals in the presence or absence of LPS challenge.** Arthritis was induced in male mice by i.p. injection of 2, 4 or 8 mg collagen II antibody cocktail (CAb) on Day 0. The effects of administering 10 μg LPS i.p. 4 days later on arthritis scores are shown in **A**, and on bodyweights in **B**. **C**, shows arthritis scores in the absence of LPS challenge and **D** shows effects on bodyweight. Data are means +/− s.e.m., n = 5-7/group.

The results of a 10 μg challenge of LPS on Day 4 are shown in Figure 2. Arthritis incidence was again 100% across all the groups. Arthritis score data were comparable for the 2 mg and 4 mg CAb groups but were rather lower than achieved for the same doses in the first experiment where a higher LPS challenge was used. However, the 8 mg CAb group showed robust disease. Weight loss for all groups between days 4 and 6 was approximately 15% before recovering and the overall condition of the animals was improved over the first experiment.

All animals in the 8 mg CAb group showed signs of arthritis before LPS challenge. This prompted an additional study where a group of animals were injected with 8 mg CAb and arthritis development followed over time without LPS challenge (Figure 2C). Once again incidence of arthritis was 100% but there was rather more variability in the data. As expected, bodyweights were maintained over the experiment (Figure 2D).

Although the study without LPS indicated that LPS was not necessary for induction of arthritis, it did appear to improve synchronization and severity of the disease and open up a good window for TNFα inhibitor studies. So we investigated whether the LPS challenge could be brought forward to a time before arthritis symptoms became manifest so that TNFα production associated with LPS challenge could be differentiated from TNFα that may be driving the arthritis.

**Figure 3:**
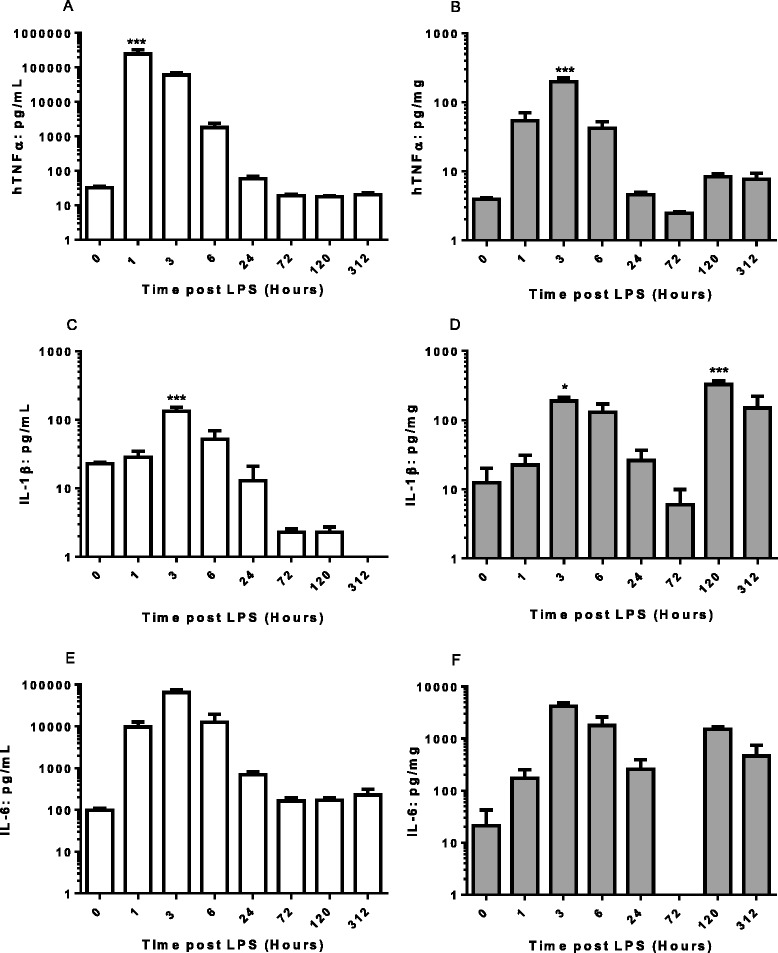
**Cytokines in serum and paws of Tg1278TNFko animals with CAIA.** Arthritis was induced in male and female mice by i.p. injection of 8 mg collagen II antibody cocktail (CAb) on Day 0 followed by 10 μg LPS i.p. 1 day later. Open bars are data for sera and closed bars are data for paws (expressed as pg cytokine/mg protein). **A** and **B** hTNFα, **C** and **D** IL-1β, **E** and **F** IL-6. Data are means +/− s.e.m., n = 3-4/group. *p < 0.05, ***p < 0.001 comparison with time 0.

We therefore measured temporal cytokine profiles in sera or joints of animals where LPS challenge was administered just 1 day after 8 mg CAb (Figure 3). When cytokines were measured in serum there was an expected acute rise in serum hTNFα following LPS challenge and this was followed by rises in IL-1β and IL-6 (Figure 3A, C and E). By 24 hours post LPS, serum cytokines were dropping back to baseline levels. There was no sign of a subsequent increase in serum cytokine levels coincident with the emergence of arthritis symptoms. Indeed, and rather curiously, there was a drop in serum IL-1β at days 3, 5 and 13 post LPS perhaps reflecting a recruitment of IL-1β secreting cells to inflamed joints (although similar reductions were not seen in circulating hTNFα or IL-6). The data for the paws were clearly biphasic with acute elevations in cytokines following LPS injection and then elevations at days 5 and 13 associated with the arthritis phase of the model (Figure 3B, D and F). These data indicated that cytokine release post LPS could be separated from cytokines associated with arthritis and in subsequent studies LPS was always administered on Day 1. This system was also used to examine the effects of the TNFα blocker etanercept.

**Figure 4:**
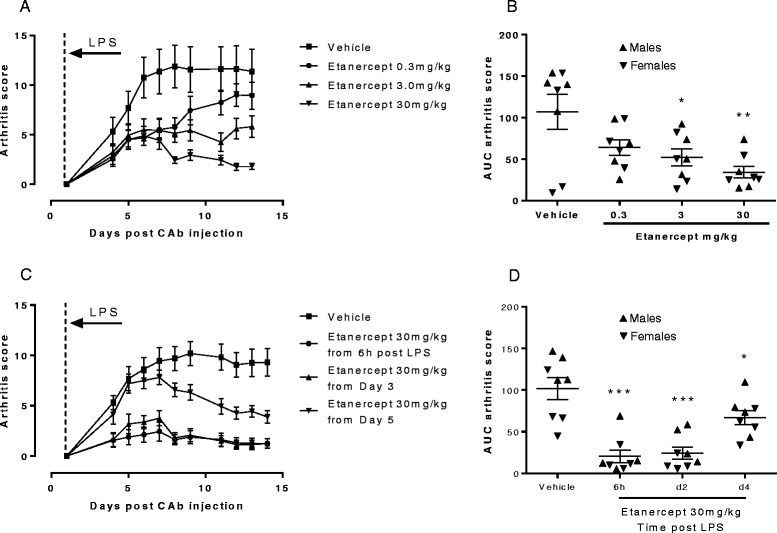
**Effects of etanercept on CAIA in Tg1278TNFko animals.** Arthritis was induced in male and female mice by i.p. injection of 8 mg collagen II antibody cocktail (CAb) on Day 0 followed by 10 μg LPS i.p. 1 day later. **A** shows the effects of dosing etanercept at 0.03, 0.3, 3 or 30 mg/kg i.v. for the first dose with subsequent doses s.c. from 6 hours post LPS administration with individual animal data expressed as area under curve (AUC) arthritis score shown in **B**. **C**, shows the effects of etanercept dosing at 30 mg/kg from 6 hours, 2 days or 4 days post LPS with individual animal data shown in **D**. Data are means +/− s.e.m., n = 8/group. *p < 0.05, **p < 0.01, ***p < 0.001 comparison with the vehicle control group.

The profile of arthritis in the Day 1 LPS challenge model was comparable to the Day 4 LPS challenge model (Figure 4A compared with Figure 2A). Other groups were dosed with etanercept at 0.3, 3 or 30 mg/kg every other day from 6 hours post LPS in order to avoid the transient TNFα spike associated with LPS challenge. The first doses were all i.v. to achieve rapid exposure to the drug and subsequent doses were s.c.. Again arthritis incidence was 100% although 2 of the 8 animals in the vehicle control group had a poor disease severity. The results are broken down further into individual animals in Figure 4B where it can be seen that both animals with mild disease were females. Nonetheless there were dose-dependent decreases in arthritis scores with etanercept treatment with 3 and 30 mg/kg treatments resulting in statistically significant reductions in arthritis scores compared with the vehicle-treated controls (p < 0.05 and p < 0.01) respectively.

**Figure 5:**
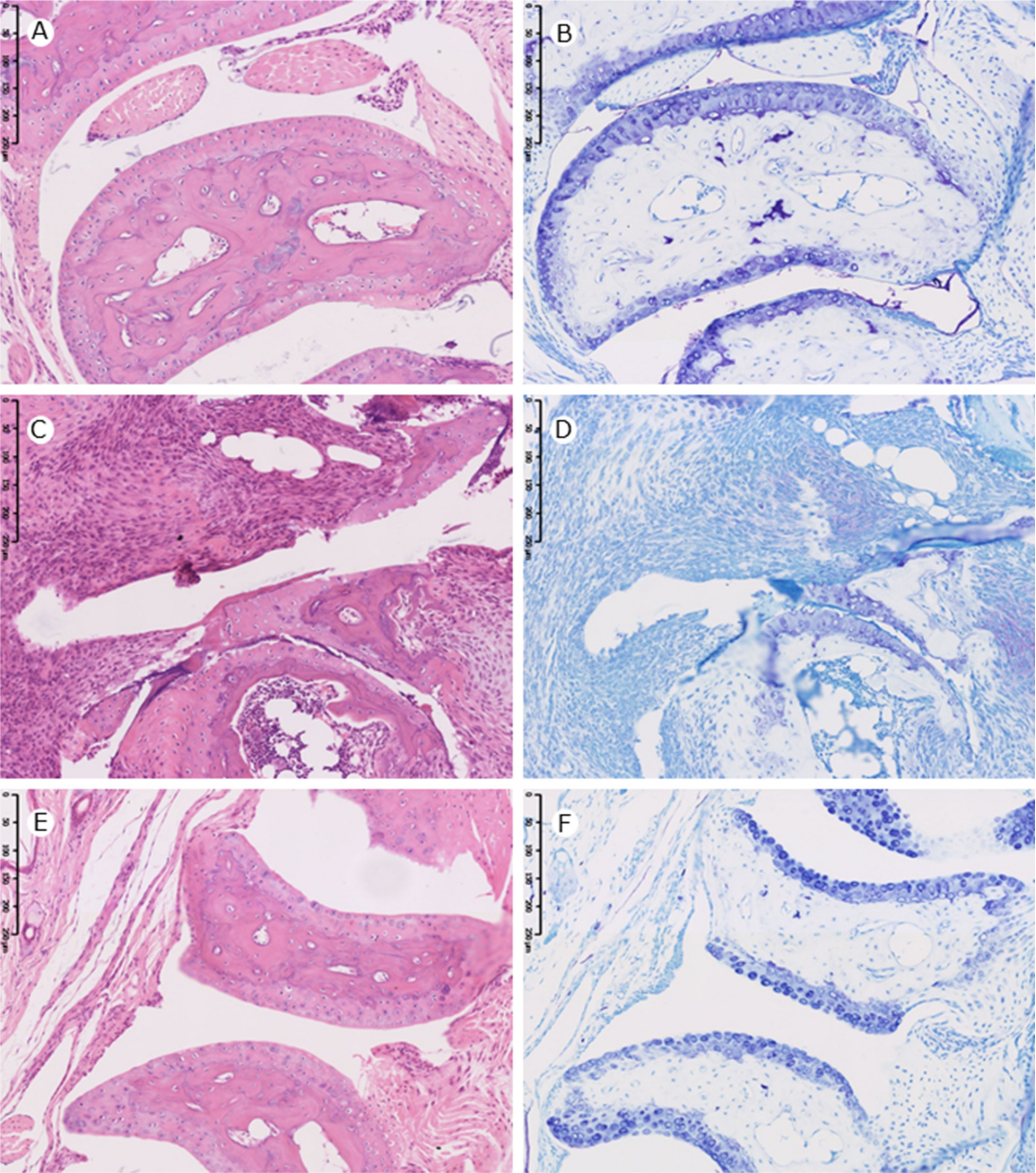
**Histology of fore paws from CAIA in Tg1278TNFko animals.** Arthritis was induced by i.p. injection of 8mg collagen II antibody cocktail (CAb) on Day 0 and 10μg LPS i.p. 1 day later. Joints were taken on Day 14. **A**, **C** and **E**, haematoxylin and eosin, **B**, **D** and **F**, toluidine blue. Compared with normal paws **(A and B)**, arthritic paws **(C and D)** show infiltration with inflammatory cells and erosion of cartilage and bone. The effect of treating with etanercept at 30mg/kg is shown in e and f. Inflammatory cell influx is reduced and there is no cartilage or bone erosion.

Joints were removed from arthritic animals on Day 14 and compared with normal joints and joints from arthritic animals that had been dosed with etanercept at 30 mg/kg i.v. 6 hours post LPS and every other day s.c. thereafter. In comparison with normal joints (Figure 5A and B) arthritic animals showed synovial hyperplasia, influx of inflammatory cells and erosion of cartilage and bone (Figure 5C and D). In etanercept-treated mice there was only mild inflammatory cell influx and no obvious destruction of cartilage and bone (Figure 5E and F).

Finally, the effect of dosing with etanercept was investigated at various times post LPS challenge (Figure 4C). Arthritis incidence in the controls was again 100% and the variability was less than in the previous experiment. However, when the data were broken down for individual animals it can be seen that the three lowest scoring animals in the vehicle control group were females (Figure 4D). Etanercept treatment at 30 mg/kg either 6 hours, 2 days or 4 days after LPS resulted in statistically significant reductions in arthritis scores compared with vehicle-treated controls (p < 0.001, p < 0.001 and p < 0.05 respectively).

## Discussion

CAIA in wild type mice is a B and T cell independent model of arthritis [13]. The main effector cells appear to be neutrophils and macrophages [14,15] that are activated by immune complexes that are formed in the joints by the binding of collagen II antibodies to the cartilage surfaces. Fc gamma receptors are important [16] as is complement activation, particularly activation through the alternative pathway [15,17]. Many of the pathological changes that are typical of RA can be seen in the joints of animals with CAIA such as synovitis, pannus formation and destruction of cartilage and bone [18,19]. The model is quick, as it requires no immune priming, and it can be performed in a variety of mouse strains including knock outs and, as here, transgenic animals. Disease onset is well-synchronized but severity of disease varies with strain and protocol [20]. The model has value for investigating effector mechanisms of arthritis and for assessing novel therapeutic agents that work on the effector stage of the disease.

The inclusion of LPS into the CAIA protocol improves disease severity but is not necessarily essential to disease induction [21]. The role that LPS plays is unclear and it probably works through multiple mechanisms that are downstream of TLR4 activation. One possibility is that in the absence of LPS, insufficient collagen II antibody is able to access the joints to promote a maximal inflammatory response and that LPS simply increases antibody access by increasing vascular permeability. But whatever the mechanism, the effects of LPS are transient, and whilst LPS may facilitate the disease process, it is not itself a part of it. For this reason it is important that potential anti-arthritic drugs should be dosed subsequent to LPS challenge because otherwise interference with LPS biology may erroneously be interpreted as an anti-arthritic effect.

In the current set of experiments we compared the standard CAIA model in wild type C57Bl/6 mice with CAIA induced in Tg1278TNFko mice with a view to developing an arthritis model that is dependent upon human TNF for the evaluation of novel therapeutic entities. The Tg1278TNFko mouse is attractive for studying human TNFα biology as TNFα production is under physiological control [4] and the animals are perfectly healthy in normal circumstances. There is no reason to believe that the mechanism of CAIA induction in Tg1278TNFko animals is fundamentally different to wild type animals. However, the replacement of the murine TNFα gene with multiple copy numbers of the human TNFα gene results in approximately 100 fold higher levels of LPS-induced TNFα than wild type animals (M. Denis manuscript in preparation), and the fact that human TNFα will only signal through murine TNFR1 and not TNFR2 [22], suggests that inflammatory TNFα biology will be accentuated. This probably explains why in our hands arthritis was more severe in the Tg1278TNFko animals than in wild type animals when an identical protocol for arthritis induction was used.

Although the protocol used to establish arthritis in wild type animals induced a more severe arthritis in Tg1278TNFko animals, the Tg1278TNFko animals did not tolerate the dose of LPS well. Weight loss was more marked than in wild type animals and even after bodyweights had started to recover the general condition of the animals was a matter for concern that required more frequent monitoring. However, it was found that the LPS challenge dose could be reduced by a factor of 10, to 10 μg, and Tg1278TNFko animals would still develop robust arthritis at the highest dose of CAb that was administered (8 mg) and that the lower LPS dose was well-tolerated. In this experiment it was also noticed that all animals in the 8 mg dose group showed signs of arthritis prior to LPS challenge. It was therefore of interest to conduct a study without LPS to examine the severity and variability of the arthritis.

A CAIA model without LPS would in fact have been the preferred model to develop but even although incidence of arthritis was again 100% the severity was low and the variability high. Whether more severe disease with less variability would be possible at higher doses of CAb is unclear but cost of the antibody cocktail, and amount of material required, make this impractical to follow up. Another possibility for an LPS-free model might be to increase the number of antibodies in the cocktail that see different collagen II epitopes, as this has been reported to increase disease severity in wild type animals [23].

Accepting that LPS challenge would be a requirement for this model we next sought to introduce the challenge earlier in the protocol i.e. one day after administration of antibody cocktail and before any signs of arthritis were manifest. This resulted in robust disease with one control animal showing a maximal score. When inflammatory cytokines were measured in the serum there was an acute rise and fall following LPS challenge but no sign of elevations in the arthritis phase of the model. By contrast, in the joints, a biphasic response was seen with elevations associated with the LPS challenge and then subsequently with the arthritis itself.

The histology of joints removed on Day 14 indicated that the arthritis induced in Tg1278TNFko was erosive as has been described for CAIA in wild type animals [18,19]. The normal joint architecture was lost, there was substantial inflammatory cell influx into the synovial tissue and there was destruction of cartilage and bone.

A good disease window (together with low data variability and high reproducibility) is desirable in a model that is to be used for inhibition studies but it is also important that the disease is not so aggressive that it is impossible to inhibit. Although the disease window is defined by the natural history of the model itself, the ability to quantify the disease process can be limiting. In this respect the arthritis scoring system is not ideal for grading therapeutic responses as it only allows scores between 0 and 4 for each paw. An extended scoring system has been suggested where numbers of involved joints are counted and assigned a score that extends from 0 to 15 for each paw [20]. However, this scoring system does not address the severity of the involved joints, and given that there will be high levels of antibody cocktail in the circulation, and that the effects of LPS are systemic, it seems likely that all joints have an equal chance of being affected by disease and that the aim of treatment in the model described here should therefore be to reduce severity of involved joints rather than number of involved joints.

Responsiveness of the model to treatment with the TNFα blocker etanercept was assessed. Treatment was initiated 6 hours after LPS challenge when the release of TNFα associated with LPS had subsided (Figure 3) and before arthritis symptoms were manifest. Doses ranged from 0.3-30 mg/kg with the first dose being administered i.v. and subsequent doses s.c every other day. Reductions in arthritis scores were dose-related and statistically significant in the 3 and 30 mg/kg groups. Furthermore etanercept treatment protected against histological changes in the joints demonstrating the utility of the model for assessing anti TNFα effects in an arthritis setting.

In a final experiment a high dose of etanercept (30 mg/kg) was dosed at various times post the LPS challenge. Dosing 6 hours after LPS was effective in preventing arthritis and confirmed the findings of the previous study. Almost as effective was dosing etanercept from 2 days after LPS. Interestingly, dosing from 4 days after LPS, when all animals were already showing signs of arthritis, was able to reverse signs and symptoms indicating that the model can be used for therapeutic dosing. The data are entirely consistent with the view that arthritis in this model is driven by human TNFα although murine factors upstream or downstream of human TNFα, or synergising with human TNFα, could also be important.

Although the disease window in this model is good, and statistically significant reductions in arthritis scores were attained with TNFα blockade, there was still some variability in the control responses. Interestingly it was female animals that were associated with the lowest disease scores in these studies. Male mice have been described as being more prone to CAIA previously but this sex difference is described as being lost after LPS treatment [20]. Whether there is a sex difference in the model described here will become clearer with time but the negative impact of variability can be addressed by adopting therapeutic dosing strategies where groups can be matched for disease severity before dosing begins. A version of the model that employs therapeutic dosing from Day 3 is now in regular use. The arthritis control and etanercept-treated control groups have been run on 5 separate occasions (at the time of manuscript submission) and the responses have proved highly reproducible between experiments.

## Conclusion

These studies describe the optimization of the CAIA model in Tg1278TNFko animals to allow the evaluation of anti-human TNFα biologics and small molecules. The main features of the model are that it is induced, that disease incidence appears to be close to 100%, arthritis scores can reach the maximal level and the model is responsive to TNFα blockade, even when the disease is well-established. These features together make the model well-suited to testing the next generation of therapeutics that will target human TNFα in rheumatoid arthritis.

## References

[1] Tak PP (2011). A personalized medicine approach to biologic treatment of rheumatoid arthritis: a preliminary treatment algorithm. Rheumatology.

[2] Raza K, Buckley CE, Salmon M, Buckley CD (2006). Treating very early rheumatoid arthritis. Best Pract Res Clin Rheumatol.

[3] Tracey KJ, Fong Y, Hesse DG, Manogue KR, Lee AT, Kuo GC, Lowry SF, Cerami A (1987). Anti-cachectin/TNF monoclonal antibodies prevent septic shock during lethal bacteraemia. Nature.

[4] Keffer J, Probert L, Cazlaris H, Georgopoulos S, Kaslaris E, Kioussis D, Kollias G (1991). Transgenic mice expressing human tumour necrosis factor: a predictive genetic model of arthritis. EMBO J.

[5] Elliott MJ, Maini RN, Feldmann M, Kalden JR, Antoni C, Smolen JS, Leeb B, Breedveld FC, Macfarlane JD, Bijl H, Woody JN (1994). Randomised double-blind comparison of chimeric monoclonal antibody to tumour necrosis factor alpha (cA2) versus placebo in rheumatoid arthritis. Lancet.

[6] Van den Berg WB, Joosten LA, Helsen MM, van de Loo FA (1994). Amelioration of established murine collagen-induced arthritis with anti-IL-1 treatment. Clin Exp Immunol.

[7] Joosten LA, Helsen MM, van de Loo FA, Van den Berg WB (1996). Anticytokine treatment of established type II collagen-induced arthritis in DBA/1 mice: a comparative study using anti-TNFa, anti-IL-1a/b, and IL-1Ra. Arthritis Rheum.

[8] Nandakumar KS, Holmdahl R (2005). Efficient promotion of collagen antibody induced arthritis (CAIA) using four monoclonal antibodies specific for the major epitopes recognized in both collagen induced arthritis and rheumatoid arthritis. J Immunol Methods.

[9] Korganow AS, Ji H, Mangialaio S, Duchatelle V, Pelanda R, Martin T, Degott C, Kikutani H, Rajewsky K, Pasquali JL, Benoist C, Mathis D (1999). From systemic T cell self reactivity to organ-specific autoimmune disease via immunoglobulins. Immunity.

[10] Ji H, Pettit A, Ohmura K, Ortiz-Lopez A, Duchatelle V, Degott C, Gravallese E, Mathis D, Benoist C (2002). Critical roles for interleukin-1 and tumour necrosis factor α in antibody-induced arthritis. J Exp Med.

[11] Kagari T, Doi H, Shimozato T (2002). The importance of IL-1β and TNFα and the non involvement of IL-6, in the development of monoclonal antibody-induced arthritis. J Immunol.

[12] Maity S, Ullanat R, Lahiri S, Shekar S, Sodhan G, Vyas A, Dyaga G, Ireni S, Nair N, Sotsios Y, Denis MC, Morawala-Patell V (2011). A non-innovator version of etanercept for treatment of arthritis. Biologicals.

[13] Pasparakis M, Alexopoulou L, Episkopou V, Kollias G (1996). Immune and inflammatory responses in TNFα-deficient mice: a critical requirement for TNFα in the formation of primary B cell follicles, follicular dendritic cell networks and germinal centers, and in the maturation of the humoral immune response. JEM.

[14] Murata K, Inami M, Hasegawa A, Kubo S, Kimura M, Yamashita M, Hosokawa H, Nagao T, Suzuki K, Hashimoto K, Shinkai H, Koseki H, Taniguchi M, Ziegler SF, Nakayama T (2003). CD69-null mice protected from arthritis with anti-type II collagen antibodies. Int Immunol.

[15] Banda NK, Hyatt S, Antonioli AH, White JT, Glogowska M, Takahashi K, Merkel TJ, Stahl GL, Mueller-Ortiz S, Wetsel R, Arend WP, Holers VM (2012). Role of C3a receptors, C5a receptors, and complement protein C6 deficiency in collagen antibody-induced arthritis in mice. J Immunol.

[16] Kagari T, Tanaka D, Doi H, Shimozato T (2003). Essential role of Fcγ receptor in anti-type II collagen antibody-induced arthritis. J Immunol.

[17] Banda NK, Takahashi K, Wood AK, Holers VM, Arend WP (2007). Pathogenic complement activation in collagen antibody-induced arthritis in mice requires amplification by the alternative pathway. J Immunol.

[18] Staines NA, Wooley PH (1994). Collagen arthritis, what can it teach us?. Br J Rheumatol.

[19] Holmdahl R, Andersson ME, Goldschmidt TJ, Jansson L, Karlsson M, Malmstrom V, Mo J (1989). Collagen induced arthritis as an experimental model for rheumatoid arthritis. Immunogenetics, pathogenesis and autoimmunity. APMIS.

[20] Nandakumar KS, Holmdahl R (2007). Collagen antibody induced arthritis. Methods Mol Med.

[21] Terato K, Harper DS, Griffiths MM, Hasty DL, Ye XJ, Cremer MA, Seyer JM (1995). Collagen-induced arthritis in mice: synergistic effect of E. coli lipopolysaccharide bypasses epitope specificity in the induction of arthritis with monoclonal antibodies to type II collagen. Autoimmunity.

[22] Lewis M, Tartaglia LA, Lee A, Bennett GL, Rice GC, Wong GHW (1991). Cloning and expression of cDNAs for two distinct murine tumor necrosis factor receptors demonstrate one receptor is species specific. Proc Natl Acad Sci U S A.

[23] Hutamekalin P, Saito T, Yamaki K, Mizutani N, Brand DD, Waritani T, Terato K, Yoshino S (2009). Collagen antibody-induced arthritis in mice: development of a new arthritogenic 5-clone cocktail of monoclonal anti-type II collagen antibodies. J Immunol Methods.

